# Chronic Alcohol Dysregulates Glutamatergic Function in the Basolateral Amygdala in a Projection-and Sex-Specific Manner

**DOI:** 10.3389/fncel.2022.857550

**Published:** 2022-04-14

**Authors:** Michaela E. Price, Brian A. McCool

**Affiliations:** ^1^Neuroscience and Alcohol Research Training Programs, Wake Forest School of Medicine, Winston-Salem, NC, United States; ^2^Department of Physiology and Pharmacology, Wake Forest School of Medicine, Winston-Salem, NC, United States

**Keywords:** presynaptic, postsynaptic, nucleus accumbens, bed nucleus of the stria terminalis, retrograde labeling

## Abstract

Chronic intermittent ethanol and withdrawal (CIE/WD) produces alcohol dependence, facilitates anxiety-like behavior, and increases post-CIE alcohol intake. The basolateral amygdala (BLA) is one of several brain regions that regulates anxiety-like behavior and alcohol intake through downstream projections to the nucleus accumbens (NAC) and bed nucleus of the stria terminalis (BNST), respectively. Previous studies revealed that CIE/WD induces input- and sex-specific adaptations to glutamatergic function in the BLA. The BLA receives information from two distinct input pathways. Glutamatergic afferents from medial structures like the thalamus and prefrontal cortex enter the BLA through the *stria terminalis* whereas lateral cortical structures like the anterior insula cortex enter the BLA through the external capsule. CIE/WD increases presynaptic glutamatergic function at *stria terminalis* synapses and postsynaptic function at external capsule synapses. Previous studies sampled neurons throughout the BLA, but did not distinguish between projection-specific populations. The current study investigated BLA neurons that project to the NAC (BLA-NAC neurons) or the BNST (BLA-BNST neurons) as representative “reward” and “aversion” BLA neurons, and showed that CIE/WD alters glutamatergic function and excitability in a projection- and sex-specific manner. CIE/WD increases glutamate release from *stria terminalis* inputs only onto BLA-BNST neurons. At external capsule synapses, CIE/WD increases postsynaptic glutamatergic function in male BLA-NAC neurons and female BLA-BNST neurons. Subsequent experiments demonstrated that CIE/WD enhanced the excitability of male BLA-NAC neurons and BLA-BNST neurons in both sexes when glutamatergic but not GABAergic function was intact. Thus, CIE/WD-mediated increased glutamatergic function facilitates hyperexcitability in male BLA-NAC neurons and BLA-BNST neurons of both sexes.

## Introduction

Chronic alcohol consumption alters reward and aversion systems in the brain, initiating a cycle of excessive alcohol consumption, abstinence/withdrawal symptoms, and relapse. Men have a higher prevalence of alcohol use disorder (AUD) than women, although this gap has been shrinking in recent years (Erol and Karpyak, 2015; Substance Abuse and Mental Health Services Administration, 2019). Men and women also differ with respect to the severity of alcohol withdrawal (WD) symptoms and the risk for alcohol-related diseases: severe WD symptoms, including seizures and delirium tremens, are more severe in men whereas women are more at risk for alcohol-related diseases, such as cardiovascular diseases, diabetes, and liver problems (Erol and Karpyak, 2015). Our laboratory and others have modeled alcohol dependence by exposing rodents to chronic intermittent ethanol (CIE) via vapor inhalation. CIE exposure is sufficient to increase anxiety-like behavior and reward thresholds during WD, as well as increase post-CIE alcohol consumption in two-bottle choice paradigms (Schulteis et al., 1995; Koob et al., 2014; Morales et al., 2015, 2018; Koob and Volkow, 2016; McGinnis et al., 2020a,b). These behavioral changes mimic symptoms observed in alcohol-dependent humans, demonstrating that the CIE paradigm induces alcohol dependence in rodents. The vast majority of preclinical studies have found that female rodents have higher baseline alcohol consumption compared to males and chronic intermittent ethanol and withdrawal (CIE/WD) only increases alcohol consumption in male rodents, suggesting that males are potentially more sensitive to changes in reward systems (Almeida et al., 1998; Vetter-O’Hagen and Spear, 2011; Morales et al., 2015; Amodeo et al., 2018; Scott et al., 2020). In contrast, WD-induced anxiety-like behavior is independent of sex (Overstreet et al., 2004; Morales et al., 2015, 2018), and thus CIE/WD alters aversion systems in a similar fashion between male and female rats.

The basolateral amygdala (BLA) has a well-established role in regulating anxiety-like behavior and alcohol consumption through its downstream projections (Läck et al., 2007, 2008; Diaz et al., 2011; McCool et al., 2014; McGinnis et al., 2020b). Glutamatergic pyramidal neurons comprise approximately 80% of BLA neurons and drive BLA output to downstream brain regions (Sah et al., 2003). Pyramidal neurons receive glutamatergic afferents from two distinct input pathways. Midline structures like the medial prefrontal cortex and the polymodal sensory thalamus send projections through the *stria terminalis* whereas more lateralized cortical structures like the anterior insula cortex project through the external capsule (Leichnetz and Astruc, 1977; McDonald et al., 1996; McDonald, 1998; Sah et al., 2003). Our laboratory has shown that inputs from the *stria terminalis* undergo presynaptic, but not postsynaptic, facilitation in CIE/WD-exposed rats (Christian et al., 2013; Morales et al., 2018; McGinnis et al., 2020b; Sizer et al., 2021). In contrast, external capsule inputs increase postsynaptic, but not presynaptic, glutamatergic function following CIE/WD (Läck et al., 2009; Christian et al., 2012; Morales et al., 2018; McGinnis et al., 2020a). These input-specific changes in glutamatergic transmission are observed in both sexes, but female rats require a longer alcohol exposure to induce the same effects (Morales et al., 2018).

Although a diverse set of inputs converge on BLA pyramidal neurons and pyramidal neurons exhibit input-specific neurophysiological changes following CIE/WD, a recent study suggests that distinct groups of BLA neurons, defined by their projection targets, receive the same set and density of inputs from upstream brain regions (Huang et al., 2021). Further, there is little collateralization between BLA projections to different downstream brain regions, indicating that BLA projections are largely independent of one another with the vast majority of BLA neurons projecting to a single downstream target (Senn et al., 2014; Beyeler et al., 2016, 2018; Huang et al., 2021). Thus, all BLA neurons integrate information from the same set of diverse sources and then BLA neurons are segregated into projection-specific groups that may independently regulate the BLA-mediated behaviors associated with each projection target. Given that most BLA neurons project to a single downstream target with little collateralization, we investigated how CIE/WD affects the neurophysiology of distinct projection-specific groups of BLA neurons.

The BLA regulates anxiety-like behavior and alcohol consumption through downstream projections to anxiety and reward-related brain regions, respectively. For example, the BLA projection to the nucleus accumbens (NAC) is preferentially responsive to reward-related stimuli, facilitates positive reinforcement, and regulates intake of rewarding substances like alcohol and sucrose (Stuber et al., 2011; Britt et al., 2012; Namburi et al., 2015; Keistler et al., 2017; Millan et al., 2017). The BLA also projects to the bed nucleus of the stria terminalis (BNST), a brain region that regulates fear and anxiety-like behavior (Kim et al., 2013; Lange et al., 2017). Inhibiting either the BLA or the BNST using optogenetics or pharmacological inactivation reduces fear and anxiety-like behavior (Walker and Davis, 1997; Läck et al., 2007; Hajizadeh Moghaddam et al., 2008; Kim et al., 2013; Sugiyama et al., 2018; Yin et al., 2019); and yet, optogenetic inhibition of the BLA projection to the BNST is anxiogenic (Kim et al., 2013). Reward and aversion are interconnected phenomena in that animals must evaluate the relative value of rewarding and anxiogenic properties in complex environments to dictate behavioral responses. For instance, the rewarding properties of stimuli may be reduced if the animal has to enter an anxiogenic or uncertain environment to receive the reward (Choi et al., 2013; Jaramillo et al., 2020; Ray et al., 2020). As such, there is some evidence to suggest that the NAC can contribute to aversive behaviors (Ray et al., 2020) and the BNST is involved in reward-related behaviors (Choi et al., 2013; Jaramillo et al., 2020) in complex environments. Thus, the predominant functions of the NAC and BNST are to regulate reward and anxiety, respectively, but can contribute to the opposing behaviors in complex environments that include rewarding stimuli and anxiogenic/uncertain environments.

In an effort to understand the projection-specific neurophysiological mechanisms underlying withdrawal-induced anxiety and increased alcohol intake, we compared how CIE/WD alters the neurophysiology of BLA neurons projecting to the NAC (BLA-NAC) and BNST (BLA-BNST) as representative “reward” and “aversion” brain regions. We found that CIE/WD enhances glutamatergic function and neuronal excitability in BLA-NAC and BLA-BNST neurons in a circuit- and sex-dependent manner. Ultimately, understanding the neurophysiological changes in these BLA projection neurons could uncover more effective pharmacotherapeutic targets aimed at reducing alcohol consumption and withdrawal-induced anxiety, as well as preventing relapse in alcohol-dependent individuals.

## Experimental Procedures

### Animals

Male and female Sprague-Dawley rats were obtained from Envigo (Indianapolis, IN). On arrival, the rats were pair-housed and given *ad libitum* access to water and standard rat chow. Animal housing rooms are on a reverse 12-h light-dark cycle (Lights OFF: 9 a.m., Lights ON: 9 p.m.). Animals were approximately 5 weeks of age (130 g) when they were surgerized and approximately 11 weeks of age when electrophysiological recordings were completed. All animal care procedures are in accordance with the National Institutes of Health *Guide for the Care and Use of Laboratory Animals* and approved by the Wake Forest Animal Care and Use Committee (WF-ACUC).

### Stereotaxic Surgeries

**FIGURE 1:**
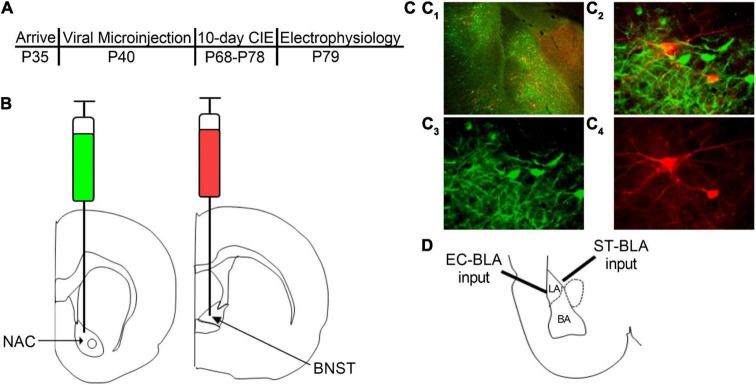
Retrograde labeling of BLA-NAC and BLA-BNST neurons. **(A)** Timeline of experimental procedures. **(B)** Schematic illustrating AAVrg-CAG-GFP into the NAC (AP + 1.80 mm, ML ± 1.5 mm, DV 6.72 mm) and AAVrg-CAG-tdTomato into the BNST (AP –0.24 mm, ML ± 0.80 mm, DV 6.45 mm). **(C_1_)** 4X overlay of GFP-expressing BLA-NAC and tdTomato-expressing BLA-BNST neurons. **(C_2_)** 40X overlay of GFP-expressing BLA-NAC and tdTomato-expressing BLA-BNST neurons. **(C_3_)** GFP expression in BLA-NAC neurons under 40 × magnification. **(C_4_)** tdTomato expression in BLA-BNST neurons under 40 × magnification. **(D)** Schematic illustrating the placement of the stimulating electrode when activating the EC-BLA and ST-BLA inputs.

The experimental timeline is as follows (Figure 1A). After an acclimation period, the rats underwent stereotaxic microinjection surgeries as described previously (McGinnis et al., 2020a). Briefly, rats were anesthetized using 3% isoflurane anesthesia and maintained under continuous 2–3% isoflurane for the remainder of the surgery with the oxygen flowmeter set at a rate of 1.0 L/min. Rats received a bilateral microinjection (0.5 μL/side) of a retrogradely transported adeno-associated viral vector expressing either GFP (AAVrg-CAG-GFP; Addgene 37825-AAVrg) into the NAC *or* tdTomato (AAVrg-CAG-tdTomato; Addgene 59642-AAVrg) into the BNST (Figure 1B). We used the following coordinates for the NAC and BNST injection sites (mm in relation to bregma): NAC (AP + 1.80, ML ± 1.50, DV 6.72) and BNST (AP −0.24, ML ± 0.80, DV 6.45). A microinjection syringe pump (Harvard Apparatus) injected the viral vectors at a rate of 0.1 μL/min for 5 min. Injectors were left in place for 10 min following the microinjection to allow the virus to diffuse into the target brain region. After completing the surgery, the incision was sutured and rats received subcutaneous injections of 3 mg/kg ketoprofen for pain management and 2 mL of warmed sterile saline. The rats were single-housed for 6–7 days until sutures were removed, after which they were pair-housed for the remainder of the experiment. In total, animals were given a 4-week period to recover and allow the virus to be transported and expressed in BLA neurons prior to CIE exposure. We confirmed injection sites by collecting coronal slices containing the NAC and BNST and visualizing postmortem GFP and tdTomato expression using fluorescence microscopy (Figure 1C). Animals with incorrect viral placement were excluded.

### Chronic Intermittent Ethanol and Withdrawal

After the 4-week recovery period, rats were exposed to CIE via vapor inhalation using our standard laboratory procedure as described previously (Morales et al., 2015, 2018; McGinnis et al., 2020a,b). Briefly, the rats were placed in custom-built plexiglass ethanol vapor chambers (Triad Plastics, Winston-Salem, NC) with their home-cages. Ethanol vapor was produced by pushing compressed air through a bubble stone submerged in 95% ethanol. The ethanol vapor was then pumped into the vapor chamber at 20–25 mg/dL. Rats were exposed to ethanol vapor for 12 h per day for 10 consecutive days, followed by a final 24-h withdrawal period. A 10-day CIE exposure and 24-h WD has been shown to model alcohol dependence in rodents by increasing anxiety-like behavior during WD and subsequently increasing alcohol intake (Morales et al., 2015, 2018; McGinnis et al., 2020a,b). Rats were weighed daily and tail blood samples were taken twice during the 10-day exposure to monitor blood ethanol concentrations (BECs; target = 150–275 mg/dL). BECs were measured using a standard, commercially available alcohol dehydrogenase/NADH (nicotinamide adenine dinucleotide plus hydrogen) enzymatic assay (Carolina Liquid Chemistries). Average BECs during the CIE exposure were 224.3 ± 6.35 mg/dL in males and 221.5 ± 9.395 mg/dL in females. Air-exposed controls were housed in similar conditions except the rats only received room air.

### Electrophysiology

#### Slice Preparation

Animals were anesthetized with isoflurane prior to decapitation, as stated in the animal care protocols approved by WF-ACUC. The brains were rapidly removed and allowed to incubate in an ice-cold sucrose-modified artificial cerebrospinal fluid (aCSF) solution containing the following (in mM): 180 sucrose, 30 NaCl, 4.5 KCl, 1 MgCl_2_ ⋅ 6 H_2_O, 26 NaHCO_3_, 1.2 NaH_2_PO_4_, 10D-glucose, and 0.10 ketamine, oxygenated with 95% O_2_/5% CO_2_, osmolarity 315–325 mmol/kg. Coronal brain slices (250 μm) containing the BLA were prepared using a VT1200/S vibrating blade microtome (Leica, Buffalo Grove, IL) and then incubated for 1-h prior to electrophysiology recordings in oxygenated, room-temperature, standard aCSF solution containing the following (in mM): 126 NaCl, 3 KCl, 2 MgSO_4_ ⋅ 7 H_2_O, 26 NaHCO_3_, 1.25 NaH_2_PO_4_, 10D-glucose, and 2 CaCl_2_ ⋅ 2 H_2_O, osmolarity 300–310 mmol/kg. All chemicals were obtained from Tocris (Ellisville, Missouri) or Sigma-Aldrich (St. Louis, MO).

#### Whole-Cell Patch-Clamp Recording

After the 1-h incubation period, BLA slices were transferred to a submersion-type recording chamber that was continuously perfused with oxygenated, room-temperature, standard ACSF at a rate of 2 mL/min. Fluorescently-labeled BLA pyramidal neurons were patched using an Olympus BZ51WI infrared differential interference contrast (IR-DIC) microscope with fluorescence attachments. Whole-cell voltage-clamp recordings were performed using recording electrodes filled with a Cs-gluconate intracellular solution containing (in mM): 145 CsOH, 10 EGTA, 5 NaCl, 1 MgCl_2_ ⋅ 6 H_2_O, 10 HEPES, 4 Mg-ATP, 0.4 Na-GTP, 0.4 QX314, 1 CaCl_2_⋅ 2 H_2_O, pH ∼7.3 (adjusted with gluconic acid), osmolarity 280–290 mmol/kg (adjusted with sucrose). Whole-cell current-clamp recordings were performed using recording electrodes filled with a K-gluconate intracellular solution containing the following (in mM): 145 K-gluconate, 10 EGTA, 5 NaCl, 1 MgCl_2_ ⋅ 6 H_2_O, 10 HEPES, 2 Mg-ATP, 0.1 Na-GTP, pH ∼7.3 (adjusted with KOH), osmolarity 280–290 mmol/kg (adjusted with H_2_O). Recording electrodes had an open-tip resistances of 4–8 MΩ. Data were acquired using an Axopatch 700B amplifier (Molecular Devices) and analyzed using pClamp 10 software (Molecular Devices). BLA principal neurons were identified based on the fluorescent label and the following electrophysiological characteristics: high membrane capacitance (>100 pF) and low access resistance (≤25 MΩ) for voltage-clamp recordings, and resting membrane potential (≤−55 mV) and action potentials that overshoot 0 mV for current-clamp recordings. Cells that did not meet these criteria were excluded from data analysis.

#### Voltage-Clamp Recordings

Glutamatergic excitatory postsynaptic currents (EPSCs), were recorded at a holding membrane potential of −65 mV and pharmacologically isolated using picrotoxin (100 μM; GABA_A_ receptor antagonist) in the bath ACSF. Glutamatergic synaptic responses were electrically evoked every 30 s by placing a concentric bipolar stimulating electrode (FHC Inc., Bowdoin, ME) within the *stria terminalis* or external capsule (Figure 1D) as indicated below. Stimulation intensities were submaximal and normalized to elicit EPSCs with amplitudes of ∼100 pA. We measured presynaptic glutamatergic function using paired electrical stimuli delivered to the *stria terminalis* and separated by a 50-ms inter-pulse interval. Using the peak amplitude of the evoked EPSCs, we calculated a paired-pulse ratio (PPR) with the following formula: (Peak Amplitude Response #2—Peak Amplitude Response #1)/Peak Amplitude Response # 1. PPR with short inter-pulse intervals are inversely related to neurotransmitter release probability (Dobrunz and Stevens, 1997). Since these results are ratios independent of amplitude, we have normalized the representative traces to the amplitude of the first EPSC to emphasize changes in PPR to emphasize treatment, sex, or projection effects on the ratios. We measured postsynaptic glutamatergic function using strontium substitution. For these recordings, calcium (2 mM) in the bath ACSF was replaced with strontium (2 mM) and a single electrical stimulus was delivered to the external capsule. Strontium is a poor substitute for calcium in terms of calcium-dependent neurotransmitter release and thus a single electrical stimulus will elicit an initial synchronous EPSC followed by prolonged asynchronous neurotransmitter release represented by unitary events arising from individual synapses (Choi and Lovinger, 1997). We measured the amplitude and inter-event interval of these asynchronous EPSCs (aEPSCs) occurring in the 400-ms window beginning 50 ms after the stimulus using MiniAnalysis software and then calculated the median amplitude and inter-event interval for each neuron. The amplitude of aEPSCs is related to AMPA receptor function and therefore postsynaptic glutamatergic function, whereas the inter-event interval of aEPSCs has an inverse relationship with calcium-independent presynaptic glutamatergic function (Choi and Lovinger, 1997).

#### Current-Clamp Recordings

Neuronal excitability and passive properties were examined in the whole-cell configuration using 600-ms current injections ranging from −100 to + 300 pA. Current step injections increased in 25 pA increments; and steps were separated by 20 s inter-trial intervals. This experiment measured passive membrane properties (resting membrane potential and input resistance), evoked action potentials, and the fast and medium afterhyperpolarization (fAHP and mAHP). Resting membrane potential was assessed prior to the first current step and input resistance was calculated as the ratio of peak voltage deflection to the amount of current injected using current steps that did not elicit an action potential. Excitability measurements were analyzed by plotting the number of action potentials elicited at each current step. The fAHP and mAHP amplitudes were evaluated using the first action potential elicited on the lowest current step for each neuron. The fAHP amplitude was defined as the difference between the action potential threshold and the peak hyperpolarization immediately following the action potential and the mAHP amplitude was the difference between the resting membrane potential and the peak hyperpolarization immediately following the end of the current step (Rau et al., 2015). We also used a 600-ms current ramp beginning at −100 pA and ending at + 300 pA. This protocol was repeated for a total of 5 trials and action potential properties were derived from the first action potential elicited for each of the 5 trials. The action potential threshold was defined as the membrane potential when the first action potential was elicited and the rheobase current was the corresponding current injected as the membrane potential reached this threshold. We also measured the amplitude and half-width of the first action potential (pClamp 10 software, Molecular Devices). These two recording paradigms were conducted under two conditions. In the first set of experiments, we measured intrinsic excitability by blocking all synaptic transmission with picrotoxin (100 μM), APV (50 μM; NMDA receptor antagonist), and DNQX (20 μM; AMPA/kainate receptor antagonist) added to the bath ACSF. In the second set, we only added picrotoxin (100 μM) to the bath ACSF.

### Statistical Analysis

Statistical analyses were conducted using Prism 9 (GraphPad Software). Data from BLA-NAC and BLA-BNST neurons were analyzed separately with 2-way ANOVAs or mixed-effects analyses, as indicated. Main effects and interactions were examined further with Bonferroni *post hoc* analyses to determine the locus of the effect. A value of *p* < 0.05 was considered to be statistically significant. All data are presented as mean ± SEM.

## Results

### Basolateral Amygdala-Nucleus Accumbens and Basolateral Amygdala-Bed Nucleus of the Stria Terminalis Neurons Can Be Identified With Retrograde Labeling and Fluorescence Microscopy

AAVrg viral particles expressing GFP or tdTomato were injected into the NAC or BNST, respectively (Figure 1B). Approximately 6 weeks after the microinjections, we examined postmortem GFP and tdTomato expression in BLA neurons (Figure 1C). The AAVrg viral vectors produced robust expression of GFP in BLA-NAC neurons and tdTomato expression in BLA-BNST neurons. BLA-NAC and BLA-BNST neurons were located primarily in the basal nucleus of the BLA and thus all of the electrophysiological recordings were directed at neurons in that subdivision (Figure 1D). All of the electrophysiology experiments were conducted after labeling either BLA-NAC or BLA-BNST neurons, not both, since the BLA projections to the NAC and BNST have very little collateralization (Huang et al., 2021).

### Chronic Intermittent Ethanol/Withdrawal Increases Glutamate Release From *Stria terminalis* Onto Basolateral Amygdala-Bed Nucleus of the Stria Terminalis Neurons

**FIGURE 2:**
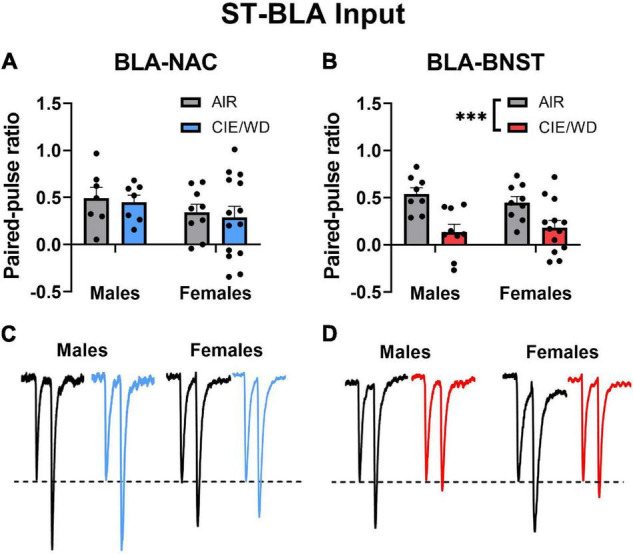
CIE/WD increased glutamate release from *stria terminalis* inputs onto BLA-BNST neurons. **(A)** CIE/WD did not affect the PPR in BLA-NAC neurons (Males: *n* = 7 AIR, 7 CIE/WD; Females: *n* = 9 AIR, 14 CIE/WD. **(B)** CIE/WD decreased the PPR in BLA-BNST neurons (Males *n* = 8 AIR, 9 CIE/WD; Females *n* = 9 AIR, 13 CIE/WD), suggesting an increase in glutamate release from *stria terminalis* inputs onto BLA-BNST neurons. **(C)** Exemplar traces from paired electrical stimuli in BLA-NAC neurons from AIR males, CIE/WD males, AIR females, and CIE/WD females (left to right). **(D)** Exemplar traces from paired electrical stimuli in BLA-BNST neurons in AIR males, CIE/WD males, AIR females, and CIE/WD females (left to right). All exemplar traces are normalized to the amplitude of the first EPSC to emphasize treatment effects on the PPR (see section “Materials and Methods”). Data analyzed by 2-way ANOVAs. ^***^p < 0.001.

After confirming that we could use retrograde labeling to identify BLA-NAC and BLA-BNST neurons, we began to characterize the effects of CIE/WD on each neuronal population. First, we examined how CIE/WD alters glutamate release probability from *stria terminalis* inputs using paired electrical stimuli to calculate the PPR, which is inversely related to neurotransmitter release probability (Dobrunz and Stevens, 1997). Several previous studies have found that CIE/WD decreases the PPR at *stria terminalis* inputs, suggesting increased glutamate release (Christian et al., 2013; Morales et al., 2018; McGinnis et al., 2020b; Sizer et al., 2021). Here, we compared CIE/WD effects on glutamate release from *stria terminalis* inputs onto BLA-NAC neurons vs. BLA-BNST neurons. A 2-way ANOVA found no main effects of CIE/WD or sex and no interaction effect on the PPR in BLA-NAC neurons (Figures 2A,C). However, in BLA-BNST neurons, a 2-way ANOVA revealed a significant main effect of CIE/WD [Figures 2B,D; *F*_(1, 35)_ = 18.74, *p* = 0.001] on the PPR with no main effect of sex or an interaction effect. The CIE/WD-induced decrease in PPR suggests that CIE/WD increases glutamate release from *stria terminalis* inputs specifically onto BLA-BNST neurons, regardless of sex.

### Chronic Intermittent Ethanol/Withdrawal Increases Postsynaptic Glutamatergic Function in External Capsule Synapses in a Circuit- and Sex-Dependent Manner

**FIGURE 3:**
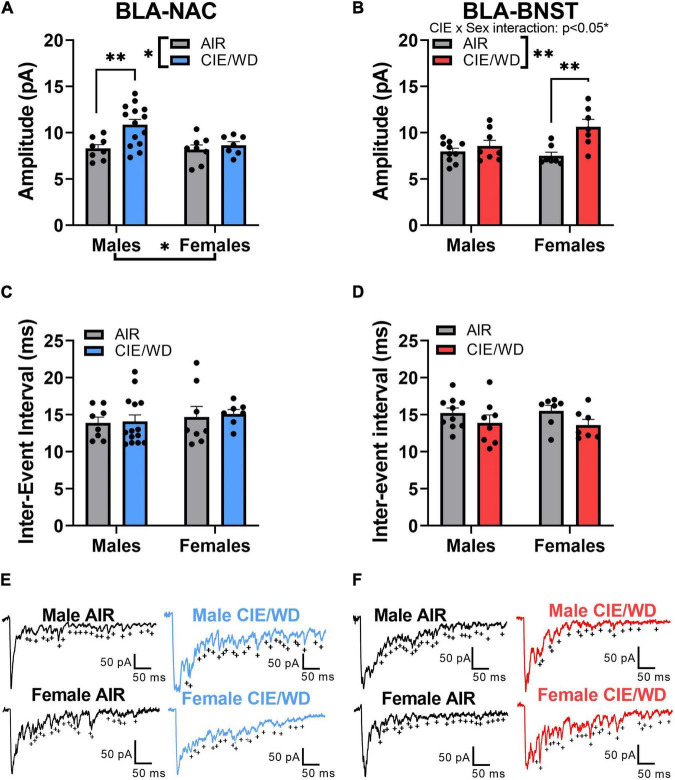
CIE/WD increased postsynaptic glutamatergic function in external capsules synapses. **(A)** CIE/WD increased aEPSC amplitude in male BLA-NAC neurons (Males: *n* = 8 AIR, 14 CIE/WD; Females: *n* = 8 AIR, 7 CIE/WD). **(B)** CIE/WD increased aEPSC amplitude in female BLA-BNST neurons (Males: *n* = 10 AIR, 8 CIE/WD; Females: *n* = 7 AIR, 7 CIE/WD). **(C)** No effect of CIE/WD on aEPSC inter-event interval in BLA-NAC neurons. **(D)** No effect of CIE/WD on aEPSC inter-event interval in BLA-BNST neurons. **(E)** Exemplar traces of aEPSCs from BLA-NAC neurons in AIR males, CIE/WD males, AIR females, and CIE/WD females (+ indicate aEPSC events). **(F)** Exemplar traces of aEPSCs from BLA-BNST neurons in AIR males, CIE/WD males, AIR females, and CIE/WD females (+ indicate aEPSC events). Data analyzed by 2-way ANOVAs. ^**^p < 0.01, *p < 0.05.

Prior studies have demonstrated that CIE/WD increases postsynaptic glutamatergic function at external capsule synapses onto the population of BLA neurons as a whole (Christian et al., 2012; Morales et al., 2018; McGinnis et al., 2020a). We examined how CIE/WD affected postsynaptic function at external capsule synapses within projection-specific populations of BLA neurons. In BLA-NAC neurons, a 2-way ANOVA found significant main effects of both CIE/WD [Figures 3A,E; *F*_(1, 33)_ = 7.073, *p* = 0.0120] and sex [*F*_(1, 33)_ = 4.162, *p* = 0.0494] on aEPSC amplitude but no significant interaction. A *post hoc* test revealed that CIE/WD increased aEPSC amplitude only in male BLA-NAC neurons (*p* = 0.0031). This suggests that CIE/WD increases postsynaptic glutamatergic function in external capsule synapses onto male BLA-NAC neurons. In BLA-BNST neurons, a 2-way ANOVA revealed a significant CIE/WD × sex interaction effect [Figures 3B,F; *F*_(1, 28)_ = 5.288, *p* = 0.0291] along with a significant main effect of CIE/WD [*F*_(1, 28)_ = 11.75, *p* = 0.0019], but no main effect of sex. *Post hoc* tests indicated that CIE/WD specifically increased aEPSC amplitude exclusively in female BLA-BNST neurons (*p* = 0.0013). Thus female BLA-BNST neurons appear more susceptible to the postsynaptic glutamatergic changes in BLA-BNST neurons compared to males.

The inter-event interval of aEPSCs is inversely related to calcium-independent presynaptic function (Choi and Lovinger, 1997). Previous data showed that neither males nor females appear to express presynaptic changes at external capsule synapses following CIE/WD (Christian et al., 2012; Morales et al., 2018; McGinnis et al., 2020a). Similar to those data, a 2-way ANOVA found no significant interaction or main effects on the aEPSC inter-event interval in either BLA-NAC neurons (Figures 3C,E) or BLA-BNST neurons (Figures 3D,F).

### Chronic Intermittent Ethanol/Withdrawal Does Not Alter the Excitability in Basolateral Amygdala-Nucleus Accumbens and Basolateral Amygdala-Bed Nucleus of the Stria Terminalis Neurons in the Absence of Synaptic Transmission

**FIGURE 4:**
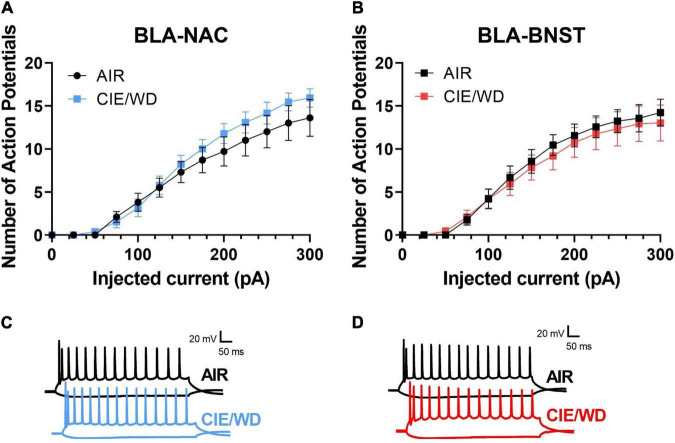
CIE/WD did not alter intrinsic excitability in male BLA neurons in the absence of synaptic transmission. **(A)** CIE/WD did not affect intrinsic excitability in male BLA-NAC neurons (*n* = 10 AIR, 13 CIE/WD). **(B)** CIE/WD did not affect intrinsic excitability in male BLA-BNST neurons (*n* = 9 AIR, 12 CIE/WD). **(C)** Exemplar traces of 600-ms current steps of –100 and + 300 pA in BLA-NAC AIR (top) and CIE/WD (bottom) neurons. **(D)** Exemplar traces of 600-ms current steps of –100 and + 300 pA in BLA-BNST AIR (top) and CIE/WD (bottom) neurons. Data analyzed by two-way ANOVAs.

Chronic ethanol alters the excitability of neurons in a variety of brain regions, including the NAC and BNST (Kash et al., 2009; Hopf et al., 2010, 2011; Marty and Spigelman, 2012; Padula et al., 2015; Pleil et al., 2015, 2016; Shan et al., 2019; Pati et al., 2020), but similar experiments have not been conducted in the BLA. To determine whether CIE/WD alters the “intrinsic” excitability (i.e., in the absence of synaptic influence) of BLA-NAC and BLA-BNST neurons in males, we first measured the number of action potentials generated by increasing postsynaptic current injections in the absence of both glutamatergic and GABAergic synaptic influence. In BLA-NAC neurons, a 2-way ANOVA showed a main effect of current injection on the number of action potentials [Figures 4A,C; *F*_(16, 336)_ = 116.0, *p* < 0.0001] but no interaction or main effect of CIE/WD. Similarly in BLA-BNST neurons, there was again a main effect of current [Figures 4B,D; *F*_(1.431, 27.18)_ = 81.67, *p* < 0.0001] but no interaction or main effect of CIE/WD. Therefore, under these recording conditions, CIE/WD had no effect on the intrinsic excitability of BLA-NAC and BLA-BNST neurons in the absence of all fast synaptic transmission.

### Chronic Intermittent Ethanol/Withdrawal Increases the Excitability in Basolateral Amygdala-Nucleus Accumbens and Basolateral Amygdala-Bed Nucleus of the Stria Terminalis Neurons Through Distinct Mechanisms When Glutamatergic Transmission Is Intact

**FIGURE 5:**
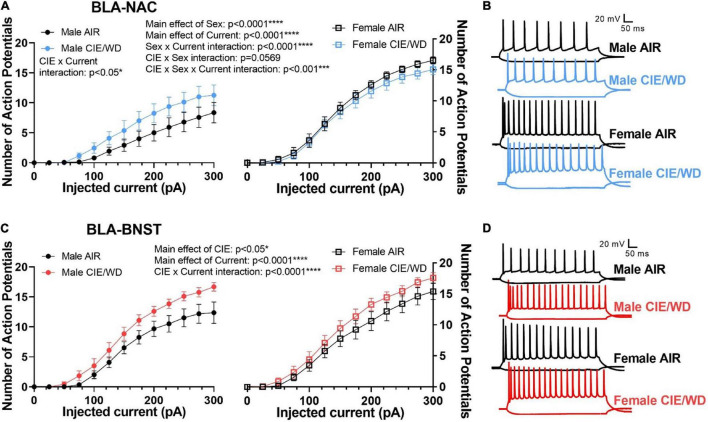
CIE/WD enhanced excitability in BLA neurons during withdrawal when glutamatergic transmission was intact. **(A)** CIE/WD increased excitability in male BLA-NAC neurons (*p* < 0.05; left) and females had higher BLA-NAC excitability than males (*p* < 0.0001; right) (Males: *n* = 15 AIR, 11 CIE/WD; Females: *n* = 13 AIR, 14 CIE/WD). **(B)** Exemplar traces of 600-ms current steps of –100 and + 300 pA in BLA-NAC neurons from AIR males, CIE/WD males, AIR females, and CIE/WD females (top to bottom). **(C)** CIE/WD increased excitability in BLA-BNST neurons, regardless of sex (*p* < 0.0001) (Males: *n* = 12 AIR, 11 CIE/WD; Females: *n* = 13 AIR, 14 CIE/WD). **(D)** Exemplar traces of 600-ms current steps of –100 and + 300 pA in BLA-BNST neurons from AIR males, CIE/WD males, AIR females, and CIE/WD females (top to bottom). Data analyzed by mixed-effects analyses.

Since CIE/WD enhanced glutamatergic function in both BLA-NAC and BLA-BNST neurons, we then investigated how this increased glutamatergic function ultimately affects the excitability of these neuronal populations when glutamatergic transmission was intact but GABAergic transmission was blocked. Using mixed-effects analysis with CIE/WD, sex, and current as main factors, we found significant main effects of sex [Figures 5A,B; *F*_(1, 49)_ = 22.41, *p* < 0.0001] and current [*F*_(1.714, 80.98)_ = 245.0, *p* < 0.0001] in BLA-NAC neurons, as well as a sex X current interaction effect [*F*_(16, 756)_ = 16.49, *p* < 0.0001] for the number of action potentials evoked by current depolarization. Although we did not find a main effect of CIE/WD, there was a significant CIE/WD X sex X current interaction effect [*F*_(16, 756)_ = 2.526, *p* = 0.0009] and a trending CIE/WD X sex interaction effect [*F*_(1, 49)_ = 3.804, *p* = 0.0569]. To determine the locus of the effect, we performed mixed-effects analyses on males and females separately. In each analysis, CIE/WD and current were the main factors. In males, the mixed-effects analysis found a significant main effect of current [*F*_(1.311, 29.18)_ = 45.90, *p* < 0.0001] and a CIE/WD X current interaction [*F*_(16, 356)_ = 1.927, *p* = 0.0172] such that CIE/WD-exposed male neurons exhibited higher BLA-NAC excitability than air-exposed controls. In contrast, there was a main effect of current [*F*_(2.072, 51.79)_ = 308.1, *p* < 0.0001] in female rats, but neither a main effect of CIE/WD nor an interaction, indicating CIE/WD did not alter BLA-NAC excitability in females. Altogether, this suggests that BLA-NAC neuron excitability is higher in females compared to males and that CIE/WD specifically increases BLA-NAC neuron excitability in males when glutamate transmission is intact but GABA transmission is blocked. Finally, we used mixed-effects analysis with CIE/WD, sex, and current as main factors to examine excitability in BLA-BNST neurons (Figures 5C,D). We found significant main effects of CIE/WD [*F*_(1, 47)_ = 5.014, *p* = 0.0299] and current [*F*_(1.732, 81.31)_ = 374.6, *p* < 0.0001], as well as a significant CIE/WD X current interaction [*F*_(16, 751)_ = 3.986, *p* < 0.0001]. These data indicate that CIE/WD increases the excitability of BLA-BNST neurons in both males and females when only glutamate transmission remained intact. Thus, CIE/WD-induced hyperexcitability is dependent on glutamate transmission and driven by the CIE/WD-induced increases in glutamatergic function described earlier (Figures 2, 3) in male BLA-NAC neurons and BLA-BNST neurons in both sexes. CIE/WD-induced hyperexcitability in these BLA projection neurons facilitates BLA output to their downstream projections, including the NAC and BNST, and therefore facilitates the behavioral changes that occur as a result of these BLA circuits being strengthened, namely increased alcohol intake and WD-induced anxiety.

**FIGURE 6:**
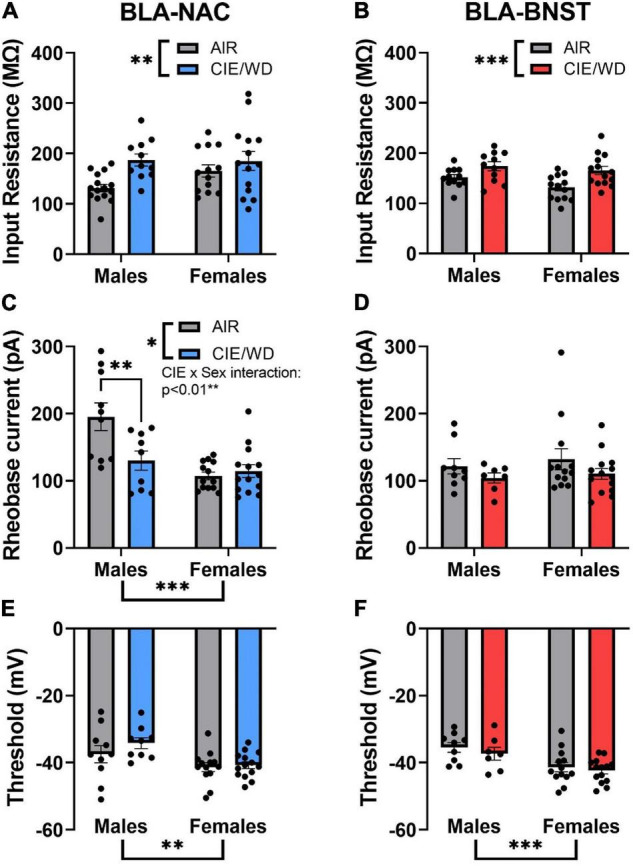
Circuit-dependent mechanisms of enhanced excitability. **(A)** CIE/WD increased input resistance in BLA-NAC neurons (Males: *n* = 15 AIR, 11 CIE/WD; Females: *n* = 13 AIR, 14 CIE/WD). **(B)** CIE/WD increased input resistance in BLA-BNST neurons (Males: *n* = 12 AIR, 11 CIE/WD; Females: *n* = 13 AIR, 14 CIE/WD). **(C)** Female BLA-NAC neurons had a lower rheobase current than males and CIE/WD decreased the rheobase current in male BLA-NAC neurons (Males: *n* = 10 AIR, 9 CIE/WD; Females: *n* = 13 AIR, 14 CIE/WD). **(D)** CIE/WD did not affect the rheobase current in BLA-BNST neurons (Males: *n* = 9 AIR, 7 CIE/WD; Females: *n* = 13 AIR, 14 CIE/WD). **(E)** Female BLA-NAC neurons had a lower action potential threshold than males (Males: *n* = 10 AIR, 9 CIE/WD; Females: *n* = 13 AIR, 14 CIE/WD). **(F)** Female BLA-BNST neurons had a lower action potential threshold than males (Males: *n* = 9 AIR, 7 CIE/WD; Females: *n* = 13 AIR, 14 CIE/WD). Data analyzed by 2-way ANOVAs. ^***^p < 0.001, ^**^p < 0.01, *p < 0.05.

To determine the mechanisms driving enhanced excitability of BLA-NAC neurons in males and BLA-BNST neurons in both sexes, we measured the input resistance, rheobase current, and action potential threshold. Input resistance is inversely related to the number of “open” ion channels on the membrane. The rheobase current is the minimum amount of current required to reach the action potential threshold and thereby elicit an action potential. 2-way ANOVAs revealed a main effect of CIE/WD on input resistance in both BLA-NAC neurons [Figure 6A; *F*_(1, 49)_ = 7.889, *p* = 0.0071] and BLA-BNST neurons [Figure 6B; *F*_(1, 46)_ = 13.41, *p* = 0.0006] such that input resistance increased in both populations. Increased input resistance in BLA-NAC and BLA-BNST neurons suggests a decrease in the number of “open” ion channels on the membranes of each population. In contrast, CIE/WD effects on the rheobase current were projection- and sex-specific. 2-way ANOVAs in BLA-NAC neurons found significant main effects of CIE/WD [Figure 6C; *F*_(1, 42)_ = 5.309, *p* = 0.0262] and sex [*F*_(1, 42)_ = 16.82, *p* = 0.0002] on the rheobase current, as well as a CIE/WD X sex interaction [*F*_(1, 42)_ = 8.270, *p* = 0.0063]. *Post hoc* analyses revealed that CIE/WD specifically decreased the rheobase current in males (*p* = 0.0032). These data indicate that females have a lower rheobase current than males in BLA-NAC neurons and only males exhibit a decrease in rheobase following CIE/WD. A decrease in rheobase indicates that the neuron requires less depolarizing current to elicit an action potential and is typically associated with greater excitability. There were no significant effects on the rheobase current in BLA-BNST neurons (Figure 6D). Interestingly, there is a significant main effect of sex on action potential threshold in both BLA-NAC [Figure 6E; *F*_(1, 42)_ = 10.28, *p* = 0.0026] and BLA-BNST [Figure 6F; *F*_(1, 39)_ = 14.81, *p* = 0.0004] neurons such that females have a lower action potential threshold than males, but no main effect of CIE/WD or any interaction. The lower action potential threshold in females is combined with a significantly lower resting membrane potential compared to males in BLA-NAC [Table 1; main effect of sex *F*_(1, 49)_ = 4.447, *p* = 0.0401] and BLA-BNST neurons [Table 1; main effect of sex *F*_(1, 46)_ = 9.632, *p* = 0.0033]. These data suggest that the lower action potential threshold may have a comparatively small impact on excitability compared to the increased input resistance and lower rheobase current.

**TABLE 1:** Cellular membrane properties.

Measure	Air-males	CIE-males	Air-females	CIE-females	*p*-values
**BLA-NAC neurons**					
Resting membrane potential (mV)	−61.13 ± 1.05	−62.92 ± 1.17	−64.25 ± 0.69	−64.33 ± 1.24	Sex *p* < 0.05
AP amplitude (mV)	91.47 ± 2.07	89.2 ± 1.16	87.14 ± 3.54	86.68 ± 1.94	N.S.
AP half-width (ms)	1.87 ± 0.06	1.95 ± 0.06	1.81 ± 0.05	1.77 ± 0.07	N.S.
**BLA-BNST neurons**					
Resting membrane potential (mV)	−61.01 ± 0.78	−59.58 ± 0.62	−62.17 ± 1.02	−64.39 ± 1.15	Sex *p* < 0.01
AP amplitude (mV)	90.39 ± 1.64	91.14 ± 2.39	92.23 ± 1.28	87.11 ± 1.76	N.S.
AP half-width (ms)	1.74 ± 0.06	1.84 ± 0.07	1.60 ± 0.03	1.71 ± 0.04	CIE *p* < 0.05, Sex *p* < 0.01

*Resting membrane potential (RMP) and the action potential amplitude and half-width of BLA-NAC and BLA-BNST neurons in the presence of picrotoxin. Values are presented as mean ± SEM. Data analyzed by 2-way ANOVAs; significant main effects are indicated along with the associated p-value. N.S., not significant.*

Next, we measured the action potential half-width and amplitude. There were no significant effects on the action potential half-width in BLA-NAC neurons (Table 1). However, a 2-way ANOVA found significant main effects of sex [Table 1; *F*_(1, 38)_ = 8.369, *p* = 0.0063] and CIE/WD [*F*_(1, 38)_ = 4.656, *p* = 0.0373] in BLA-BNST neurons, suggesting that CIE/WD increases the action potential half-width and males have a larger half-width compared to females. Inactivating calcium-activated potassium channels, specifically big-conductance BK channels, broaden the action potential half-width in the lateral amygdala (Faber and Sah, 2003). BK channels hyperpolarize the membrane after an action potential, so BK channel inactivation would reduce the rate of hyperpolarization and therefore broaden the action potential half-width. These findings suggest that CIE/WD may inactivate BK channels and that there are fewer active BK channels in males compared to females. There were no significant effects of CIE/WD or sex on the action potential amplitude in either group of neurons (Table 1).

**FIGURE 7:**
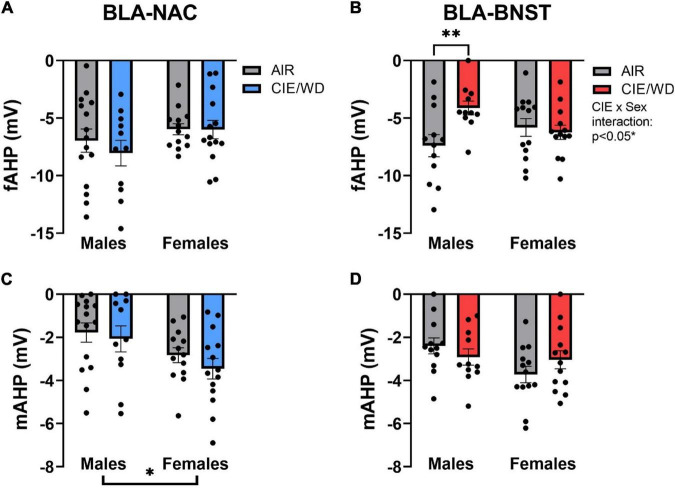
Ethanol-induced and sex-dependent effects on afterhyperpolarization in BLA neurons. **(A)** CIE/WD did not alter the fAHP amplitude in BLA-NAC neurons (Males: *n* = 15 AIR, 11 CIE/WD; Females: *n* = 13 AIR, 14 CIE/WD). **(B)** CIE/WD reduced the fAHP amplitude only in male BLA-BNST neurons (Males: *n* = 12 AIR, 11 CIE/WD; Females: *n* = 13 AIR, 14 CIE/WD). **(C)** Female BLA-NAC neurons had a larger mAHP amplitude compared to males (Males: *n* = 15 AIR, 11 CIE/WD; Females: *n* = 13 AIR, 14 CIE/WD). **(D)** CIE/WD did not alter the mAHP amplitude in BLA-BNST neurons (trending sex effect *p* = 0.0688) (Males: *n* = 12 AIR, 11 CIE/WD; Females: *n* = 13 AIR, 14 CIE/WD). Data analyzed by 2-way ANOVAs. ^**^p < 0.01, *p < 0.05.

Afterhyperpolarization can also impact excitability by hyperpolarizing the neuron and preventing it from returning to resting membrane potential (Guo et al., 2012; Sun et al., 2015). In doing so, the neuron is unable to reach the action potential threshold for a longer period of time and thereby reduces the number of action potentials generated in a set timeframe. The fAHP is the hyperpolarization peak immediately following an action potential whereas the mAHP is the hyperpolarization peak immediately following the end of a depolarizing current step injection (Rau et al., 2015). Although there were no significant effects on the fAHP in BLA-NAC neurons (Figure 7A), there was a significant CIE/WD X sex interaction [Figure 7B; *F*_(1, 45)_ = 5.974, *p* = 0.0185] and a trending CIE/WD effect [*F*_(1, 45)_ = 3.604, *p* = 0.0640] in BLA-BNST neurons. *Post hoc* analyses illustrated that CIE/WD reduced the fAHP amplitude only in male BLA-BNST neurons (*p* = 0.0093). BK channels contribute to the fAHP, as well. BK channel inactivation would reduce fAHP amplitude and increase the number of action potentials produced (Guo et al., 2012; Sun et al., 2015), as observed in male BLA-BNST neurons following CIE/WD exposure. In contrast, CIE/WD did not alter the mAHP in either population of BLA neurons, but there is a significant main effect of sex on the mAHP amplitude in BLA-NAC neurons [Figure 7C; *F*_(1, 49)_ = 6.736, *p* = 0.0124] and a trending effect of sex in BLA-BNST neurons [Figure 7D; *F*_(1, 45)_ = 3.476, *p* = 0.0688]. In both cases, females tended to have a larger mAHP amplitude compared to males. Another calcium-activated potassium channel, small-conductance SK channels, contribute to the mAHP (Hopf et al., 2010; Shan et al., 2019). This indicates that females may have a greater number of active SK channels, producing a larger mAHP amplitude. Typically, larger mAHP amplitudes reduce excitability, but the larger mAHP amplitude in female BLA neurons appears to be insufficient to decrease excitability here.

Altogether, BLA-NAC and BLA-BNST neurons both expressed increased input resistance following CIE/WD, suggesting the treatment may close some ion channel. Given that increased input resistance is associated with greater excitability of these neurons, CIE/WD seems to down-regulate a hyperpolarizing current like those carried by BK and SK channels. Evidence suggests that inhibiting either BK or SK channels increased input resistance, enhanced excitability, and reduced AHP amplitude (Nelson et al., 2003). Moreover, inhibiting BK channels occluded hyperpolarization-induced firing rate potentiation in the medial vestibular nucleus (Nelson et al., 2003). In BLA-BNST neurons, CIE/WD-induced hyperexcitability and increased input resistance may be mediated by the inactivation of BK channel currents. This is supported by a larger action potential half-width and a male-specific reduction in fAHP amplitude. There are potential sex differences in these effects as CIE/WD does not alter fAHP amplitude in females, suggesting other potassium channels could be closing to facilitate CIE/WD-induced hyperexcitability or that BK channels have less of an impact on fAHP amplitude in females. In BLA-NAC neurons, CIE/WD-induced hyperexcitability and increased input resistance are not associated with changes in action potential half-width, fAHP, or mAHP, indicating neither BK or SK channels are involved. Rather, BLA-NAC neurons are heavily influenced by lower rheobase currents as females have a lower rheobase compared to males and there is a male-specific reduction in rheobase, both of which mirror the higher BLA-NAC excitability in females compared to males and CIE/WD-induced hyperexcitability in males.

## Discussion

The current study demonstrates that BLA-NAC and BLA-BNST neurons express both circuit- and sex-dependent neurophysiological changes following CIE/WD, suggesting that CIE/WD may differentially impact BLA projections to these brain regions. In BLA-NAC neurons, CIE/WD enhances postsynaptic glutamatergic function and excitability specifically in males. By comparison, the effects of CIE/WD on glutamatergic function and excitability of BLA-BNST neurons are mostly independent of sex. CIE/WD increases glutamate release and excitability in both sexes with an additional female-specific increase in postsynaptic glutamatergic function. Altogether, enhanced glutamatergic function in male BLA-NAC neurons and BLA-BNST neurons in both sexes drives CIE/WD-induced hyperexcitability in those same neurons, as these changes in excitability are absent when glutamatergic transmission is blocked. CIE/WD-induced hyperexcitability in male BLA-NAC neurons and BLA-BNST neurons in both sexes can then facilitate increased alcohol intake and anxiety-like behavior through the BLA-NAC and BLA-BNST projections, respectively.

### Basolateral Amygdala-Nucleus Accumbens Projection Neurons

In BLA-NAC neurons, CIE/WD increases postsynaptic glutamatergic function at external capsule synapses but did not alter presynaptic function at these synapses nor at *stria terminalis* inputs. Interestingly, the increased postsynaptic glutamatergic function is only observed in males and facilitates a male-specific increase in BLA-NAC excitability, driven by increased input resistance and a male-specific decrease in the rheobase current. Female BLA-NAC neurons are more excitable at baseline compared to males due to a lower rheobase current and action potential threshold, but do not become more excitable after CIE/WD. These data appear to parallel behavioral studies illustrating that females consume more alcohol at baseline compared to males and that only males increase alcohol consumption after CIE/WD (Morales et al., 2015). Altogether, behavioral and electrophysiological experiments suggest that chronic ethanol strengthens the excitability of reward circuits in males, leading to increased alcohol consumption, and that females may be protected from CIE/WD-increased alcohol consumption due to high baseline excitability in BLA-NAC neurons.

Enhancing postsynaptic glutamatergic function and excitability in male BLA neurons projecting to the NAC could translate to greater glutamate release from BLA inputs onto NAC neurons following CIE/WD. BLA inputs onto NAC neurons have a relatively low glutamate release probability in naïve male mice (Britt et al., 2012) which may allow for a more dynamic response to CIE/WD exposure. There is considerable evidence that chronic alcohol exposure enhances the excitability of GABAergic medium spiny neurons in the NAC core of male rodents, the predominant neuron type in the NAC (Hopf et al., 2010, 2011; Shan et al., 2019). This is driven by down-regulation of calcium-activated potassium channels that normally suppress neuronal excitability by hyperpolarizing the cell membrane. Specifically, chronic ethanol enhances excitability by reducing SK channel currents, SK-mediated mAHP amplitude, and SK3 subunit protein expression in NAC core MSNs (Hopf et al., 2010; Shan et al., 2019). CIE/WD produces a male-specific increase in excitability and increased input resistance in BLA-NAC neurons, indicating a decrease in the number of “open” ion channels delivering hyperpolarizing current, as well as a male-specific decrease in the rheobase. There were no CIE/WD-induced changes to the action potential half-width, fAHP amplitude, or mAHP amplitude, suggesting BK and SK channel inactivation are not the cause of CIE/WD-mediated hyperexcitability in male BLA-NAC neurons. BK channel inactivation is associated with broader action potential half-width and reduced fAHP amplitude (Faber and Sah, 2003; Guo et al., 2012; Sun et al., 2015). SK channel inactivation is associated with reduced mAHP amplitude (Hopf et al., 2010; Shan et al., 2019). Another type of potassium channel is likely driving the increased excitability in male BLA-NAC neurons.

Our findings also show that female BLA-NAC neurons have greater basal excitability compared to males. This may produce a “ceiling effect” and act as “protective” against the CIE/WD-dependent increases in excitability that was observed in male BLA-NAC neurons. Although it is unclear whether NAC MSNs also have greater basal excitability in females compared to males, greater basal excitability in female BLA-NAC neurons is consistent with previous reports that female BLA neurons in general have a higher basal firing rate and are more sensitive to glutamate-driven increases in firing rate compared to males (Blume et al., 2017). Greater basal excitability in female BLA-NAC neurons could ultimately produce greater glutamate release from BLA inputs onto female NAC MSNs. However, to our knowledge, there are no published studies on sex differences in glutamate release from BLA inputs onto NAC neurons in naïve or ethanol-exposed animals. Despite the absence of CIE/WD-mediated effects on mAHP amplitudes, we found that the mAHP amplitude is larger in female BLA-NAC neurons compared to males, suggesting there may be sex differences in SK channels. Recent studies have demonstrated sex differences in the effects of SK channel pharmacological inhibitors following chronic stress in other brain regions like the dorsal raphe (Oliver et al., 2020). Similarly, the mAHP amplitude is sex-dependent in the entorhinal cortex layer VI, leading to sex differences in neuronal excitability (Chung and Bailey, 2019). These differences in SK channel function may be due to direct regulation by sex hormones like estrogen or progesterone. For instance, 17β-estradiol alters SK3 subunit mRNA expression in female gonadotropin-releasing hormone-expressing neurons (Bosch et al., 2013).

### Basolateral Amygdala-Bed Nucleus of the Stria Terminalis Projection Neurons

In BLA-BNST neurons, CIE/WD has profound effects on glutamatergic function at *stria terminalis* and external capsule synapses in both sexes. For example, CIE/WD augments glutamate release from *stria terminalis* inputs, independent of sex. Female BLA-BNST neurons also express increased postsynaptic glutamatergic function at external capsule synapses while male neurons with the same projection do not. This increased glutamatergic function may complement the CIE/WD-enhanced excitability in both male and female BLA-BNST neurons. Thus, the BLA to BNST “aversion” circuit is potentiated after CIE/WD, regardless of sex. These data parallel behavioral studies showing that chronic ethanol increases withdrawal-induced anxiety-like behavior in both sexes (Morales et al., 2015, 2018).

Although both male and female BLA-BNST neurons express enhanced excitability after CIE/WD, the mechanisms responsible for producing this outcome may be at least partially sex-dependent. CIE/WD increased the input resistance in both males and females, suggesting a loss of “open” channels carrying hyperpolarizing current like BK and SK channels. BK channel inactivation reduces the fAHP amplitude, broadens the action potential half-width, and increases input resistance (Faber and Sah, 2003; Nelson et al., 2003; Guo et al., 2012; Sun et al., 2015). Both acute restraint stress and fear conditioning enhance excitability in the male lateral amygdala neurons by reducing fAHP amplitude and BK channel expression (Guo et al., 2012; Sun et al., 2015). CIE/WD similarly reduced fAHP amplitude but only in male BLA-BNST neurons. This suggests that CIE/WD could decrease BK channel contributions in a sex-specific fashion. Although input resistance was also increased in female BLA-BNST neurons, neither the fAHP nor the mAHP were altered by CIE/WD. The channels responsible for this outcome in females are not currently known.

Our data suggest that CIE/WD increases BLA-BNST neuron excitability, a circuit that regulates anxiety-like behavior. Classic pharmacological studies have consistently shown that activity in both the BLA and BNST generally drive conditioned fear and anxiety-like behavior (Walker and Davis, 1997; Läck et al., 2007; Hajizadeh Moghaddam et al., 2008; Kim et al., 2013; Sugiyama et al., 2018; Yin et al., 2019). However, recent optogenetic studies have suggested this may be an overly simplistic view of the BLA-BNST circuit (Kim et al., 2013; Lange et al., 2017). So it is not clear whether the neurophysiological changes described here are directly representative of and help drive CIE/WD-enhanced anxiety behaviors or if they represent a compensatory mechanism acting in opposition to such outcomes. Further, local circuits within the BNST may complicate our understanding of functional contributions by BLA inputs to that brain region. For example, the BLA projects to two distinct subpopulations in the lateral central amygdala (CeA): SOM^+^ /PKCδ^–^ neurons and SOM^–^/PKCδ^+^ neurons. In naïve animals, the excitatory strength of BLA-CeA^SOM–^ synapses is stronger than BLA-CeA^SOM+^ synapses, decreasing expression of anxiety related behavior (Tye et al., 2011; Li et al., 2013; Cai et al., 2014; Janak and Tye, 2015). Fear conditioning reverses the relative strength of these synapses by specifically increasing glutamate release from the BLA onto CeA^SOM+^ neurons and decreasing glutamate release onto CeA^SOM–^ neurons (Li et al., 2013), ultimately increasing anxiety. Given the neuronal and anatomical heterogeneity of the BNST, CIE/WD may cause a similar shift in synaptic strength from one BNST population to another such that the BLA-BNST projection is anxiolytic in naïve animals and anxiogenic in CIE/WD-exposed animals. Future behavioral experiments are critical to untangling the BLA-BNST circuitry responsible for regulating anxiety in naïve and CIE/WD-exposed animals.

### Sex: Positive and Negative Reinforcement

Altogether, chronic ethanol impacts BLA-NAC neurons in males but not females; and, although there are substantial effects on BLA-BNST neurons in both sexes, females have additional neurophysiological changes that the males do not. Female BLA-BNST neurons have both pre- and postsynaptic facilitation at *stria terminalis* and external capsule synapses, respectively. This disparity suggests that male rodents may be more driven primarily by positive reinforcement following CIE/WD while females may be more driven by negative reinforcement. Clinical studies have illustrated that adolescent boys are significantly more likely to initiate first-time alcohol consumption for alcohol enhancement motives (i.e., drinking to have fun) whereas adolescent girls are twice as likely to initiate drinking as a coping mechanism for negative emotions (Kuntsche and Müller, 2011; Dir et al., 2017). Furthermore, binge drinking was associated with higher impulsivity/sensation-seeking in young adult men and higher neuroticism/anxiety in young adult women (Adan et al., 2016; Dir et al., 2017). Thus, it is imperative that we consider the motivations behind risky drinking behaviors to develop pharmacotherapies that effectively target reward-seeking and negative affect for men and women, respectively.

### Input vs. Output Pathway Contributions

The BLA receives afferents from numerous brain regions, including but not limited to, the prefrontal cortex, temporal and insular regions, thalamus, and hippocampus (Leichnetz and Astruc, 1977; McDonald et al., 1996; McDonald, 1998; Sah et al., 2003; Huang et al., 2021). The projection targets of the BLA are equally diverse, including but not limited to, the NAC, BNST, CeA, prefrontal cortex, and hippocampus (Senn et al., 2014; Janak and Tye, 2015; Beyeler et al., 2016, 2018; Huang et al., 2021). A recent study utilized a novel approach to examine BLA input-output relationships with a specific focus on the collateralization of BLA projections and input diversity to projection-specific BLA neuron subpopulations (Huang et al., 2021). Importantly, there is little collateralization between BLA projections to the NAC and BNST; and yet, the inputs to BLA-NAC and BLA-BNST neurons are indistinguishable. These findings, in the context of our study, highlight the specificity of CIE/WD effects on BLA-NAC and BLA-BNST neurons and may suggest that BLA-resident mechanisms may govern input-specific synaptic alterations. Although CIE/WD ultimately enhanced excitability in both groups of neurons, the mechanisms by which this occurred were distinct despite these populations receiving anatomically identical inputs.

## Conclusion

Our findings are the first to illustrate projection-specific alterations in BLA neurophysiology following CIE/WD. BLA-mediated behaviors, including anxiety and alcohol consumption, are modulated through downstream projections to anxiety and reward-related regions like the BNST and NAC, respectively. BLA-NAC (“reward”) neurons exhibit postsynaptic facilitation of glutamatergic function and enhanced excitability exclusively in CIE/WD-exposed males. Moreover, female BLA-NAC neurons have higher basal excitability compared to males. The striking parallels between the baseline sex differences and CIE/WD-induced effects on BLA-NAC excitability and alcohol intake strongly suggest that neurophysiological changes in BLA-NAC neurons directly modulate processes governing alcohol intake. CIE/WD effects in BLA-BNST (“aversion”) neurons are largely independent of sex as they exhibit presynaptic facilitation of glutamatergic function and increased excitability in both sexes, as well as postsynaptic facilitation of glutamatergic function in females. The parallels between these neurophysiological outcomes and anxiety-like behavior following CIE/WD strongly suggest that BLA-BNST neurons modulate anxiety-like behavior. Further experiments will be necessary to fully elucidate the mechanisms by which females are “protected” from CIE/WD-induced changes to glutamatergic function and excitability. In addition, future experiments could explore additional BLA projections related to anxiety and reward-related behaviors, such as projections to the CeA, hippocampus, and prelimbic cortex, as well as the intrinsic interactions between these distinct populations of BLA neurons.

## Data Availability Statement

The original contributions presented in the study are included in the article/supplementary material, further inquiries can be directed to the corresponding author/s.

## Ethics Statement

The animal study was reviewed and approved by the Wake Forest School of Medicine ACUC.

## Author Contributions

MP helped with the experimental design, performed all experiments and data analysis, and wrote the manuscript. BM conceived the project, helped with the experimental design, and assisted with the experimental execution, and manuscript writing in an editorial and advisory capacity. Both authors contributed to the article and approved the submitted version.

## Conflict of Interest

The authors declare that the research was conducted in the absence of any commercial or financial relationships that could be construed as a potential conflict of interest.

## Publisher’s Note

All claims expressed in this article are solely those of the authors and do not necessarily represent those of their affiliated organizations, or those of the publisher, the editors and the reviewers. Any product that may be evaluated in this article, or claim that may be made by its manufacturer, is not guaranteed or endorsed by the publisher.
